# Significance of Micrometastases: Circulating Tumor Cells and Disseminated Tumor Cells in Early Breast Cancer

**DOI:** 10.3390/cancers2021221

**Published:** 2010-06-08

**Authors:** Catherine Oakman, Marta Pestrin, Silvia Bessi, Francesca Galardi, Angelo Di Leo

**Affiliations:** 1 ‘Sandro Pitigliani’ Medical Oncology Unit, Department of Oncology, Hospital of Prato, Istituto Toscano Tumori, Piazza Ospedale 2, 59100, Prato, Italy; E-Mail: coakman@usl4.toscana.it (C.O.); 2 Translational Research Unit, Hospital of Prato, Istituto Toscano Tumori, Piazza Ospedale 2, 59100, Prato, Italy

**Keywords:** breast cancer, adjuvant, micrometastases, circulating tumor cells, disseminated tumor cells

## Abstract

Adjuvant systemic therapy targets minimal residual disease. Our current clinical approach in the adjuvant setting is to presume, rather than confirm, the presence of minimal residual disease. Based on assessment of the primary tumor, we estimate an individual’s recurrence risk. Subsequent treatment decisions are based on characteristics of the primary tumor, with the presumption of consistent biology and treatment sensitivity between micrometastases and the primary lesion. An alternative approach is to identify micrometastatic disease. Detection of disseminated tumor cells (DTC) in the bone marrow and circulating tumor cells (CTC) from peripheral blood collection may offer quantification and biocharacterization of residual disease. This paper will review the prognostic and predictive potential of micrometastatic disease in early breast cancer.

## 1. Introduction

In the adjuvant setting, following definitive surgery for early stage breast cancer, patients are free of overt disease. The aim of adjuvant therapy is eradication of residual microscopic malignancy based on the concept that residual disease is the source of subsequent incurable systemic relapse. However, with current tools, the presence of minimal residual disease is presumed, not measured. 

Our current approach for determining adjuvant systemic therapy is to assess the primary tumor using traditional clinical and pathological features (e.g., young patient age, high tumor grade, large tumor size, lymph nodal involvement, lack of hormone receptor (HR) expression, and overexpression of HER2) or more recent molecular profiling tools (e.g., 21-gene OncotypeDx and 70-gene Mammaprint). Based on assessment of the primary tumor, risk of disease recurrence is estimated. The estimates are based on breast cancer relapse and overall survival data from prior clinical trials. Furthermore, following the presumption of the presence of residual disease, subsequent treatment decisions are based on characteristics of the primary tumor (e.g., HR, HER2), with the presumption that biological characteristics and treatment sensitivity are consistent between the primary tumor and micrometastases. There are no current tools in the adjuvant setting to assess treatment efficacy once a treatment has commenced. See Table 1.

**Table 1:** Early breast cancer: presumptions made in the adjuvant setting regarding the presence and characteristics of micrometastases.

**Current presumptions in the adjuvant setting:**
1. Presence of residual disease
2. Consistent biological characteristics between residual disease and the primary tumor
3. Consistent treatment sensitivity between residual disease and the primary tumor

Using such an approach has limitations. Some individuals with low risk disease develop recurrence despite treatment. Some individuals assessed as high risk remain relapse free in the long-term without any systemic adjuvant therapy. 

Such risk overestimation by traditional features is exemplified by the pivotal adjuvant chemotherapy trial in which 386 patients assessed as high risk, based on nodal involvement, were randomized to either a surgery plus cyclophosphamide, methotrexate, and 5-fluorouracil trial (CMF) or to surgery alone [1]. Thirty-year follow-up data confirms a sustained survival benefit in the chemotherapy arm over surgery alone (relapse free survival (RFS): 29% *vs.* 22%). Important to note is that 22% of patients had an excellent outcome despite apparent high-risk disease and no systemic treatment. Similarly, in a 20-year follow-up of 90 patients, with ER negative, node-negative disease, randomized to surgery plus CMF *versus* surgery alone, chemotherapy was associated with sustained benefit (approximate RFS: 65% *vs.* 45%) [1]. Again, substantial RFS was seen in a substantial number of patients treated with surgery alone.

It may be thought that such risk overestimation may be overcome by recent molecular profiling tools. However, these tools are also shown to overestimate risk in a substantial number of women. The 21-gene Oncotype Dx was assessed in 355 placebo-treated patients from the NSABP-B14 trial, all with node-negative, ER positive disease [2]. Ten-year distant-recurrence free survival (DRFS) for these patients treated with surgery alone was 86%, 62% and 69% for low, intermediate, and high recurrence scores, respectively. For women with a high recurrence score, it would be reasonable, based on current data, to offer adjuvant systemic chemotherapy in addition to endocrine therapy. However, nearly 70% of women with a high recurrence score had long-term DRFS without any adjuvant intervention. Similarly, one study from the Netherlands Cancer Institute Tissue Bank applied the 70-gene Mammaprint to 151 lymph node-negative patients, only 10 of whom received any adjuvant therapy. The research showed a 10-year distant metastases free survival of 87% for good signature patients and 44% for the poor prognosis cohort [3]. 

A striking feature of these current assessment tools in the adjuvant setting is overestimation of risk of disease recurrence. Some individuals, despite apparent ‘high risk’ disease, clearly have excellent long-term disease-free outcomes. Refined assessment, with identification of these individuals, would spare them unnecessary and potentially toxic therapy.

## 2. Micrometastases

A promising alternative to presumption of residual disease is actual measurement of micrometastases. We now have tools to identify disseminated tumor cells (DTC) in the bone marrow and circulating tumor cells (CTC) from the peripheral blood. 

Emerging evidence for the clinical potential of micrometastases in breast cancer is reflected in their consideration within recent guidelines. Both the American Society of Clinical Oncology 2007 Recommendations for the use of Tumor Markers in Breast Cancer and the San Gallen Consensus of 2009 reviewed evidence for micrometastases [4,5]. Whilst recognizing available data, especially for the strong prognostic role of DTC, and acknowledging the future potential of these tools, the guidelines advise that available evidence and methodology are insufficient to currently support a routine role in the management of patients with breast cancer.

Detection of micrometastases may have future value in early breast cancer for refining prognoses, monitoring treatment efficacy, and allowing biocharacterization of residual disease. See Table 2.

**Table 2:** Detection of micrometastases: potential roles in early breast cancer.

**Potential roles for micrometastases:**
1. Refine prognosis
2. Serial detection to assess efficacy of adjuvant therapy
3. Biocharacterization of residual disease, with potential therapeutic implications

## 3. Detection

Detection of DTC and CTC is largely based on their status as epithelial cells and the normal lack of epithelial cells in the bone marrow and peripheral circulation. In samples from breast cancer patients, especially in patients with early disease, the occurrence of DTC or CTC is a rare event. The numbers quoted are in the order of one cancer cell per 1-10 million normal cells. Obviously, isolation of these cells is a tremendous technical challenge. DTC and CTC may be identified using a variety of immunohistochemistry (IHC) and molecular techniques.

### 3.1. Detection of DTC

DTC in breast cancer patients are defined as epithelial cells found in a bone marrow aspirate that may or may not be breast derived, malignant, or viable [6]. The most frequent method of detection is IHC staining for epithelial cells in bone marrow aspirates following cytospin, using a pancytokeratin monoclonal antibody, such as A45-B/B3 (recognizing several cytokeratin epitopes CK8, CK18 and CK19) or AE1/AE3 (detecting CK5, CK7, CK8, and CK19). Using IHC for DTC detection, the sensitivity ranges from 1 DTC in 10^5^ to 1 in 10^6^ leucocytes, with DTC detected in about 30% of early breast cancer patients [7].

Recent guidelines recommend that a positive DTC result is the presence of at least one CK+ cell in the bone marrow which meets morphological criteria for malignancy [8]. These morphological features include large cells with a large nucleus, nuclear granulation or stippling, a large nucleolus, strong or irregular staining for cytokeratin, cytokeratin filaments, a high nuclear to cytoplasmic ratio, and the presence of cell clusters. Morphological assessment distinguishes CK+ cells as DTC, haemopoietic, squamous, or normal epithelial cells. Whilst epithelial cells are rarely found in the bone marrow, they have been reported in 1–2% of normal volunteers [9]. With morphology, DTC rates are approximately 13–15% [6].

Other methods with the potential for increased accuracy of DTC detection include antibody-linked immunomagnetic enrichment, flow cytometry, PCR, and RT-PCR arrays.

### 3.2. Detection of CTC

Since the mid 19^th^ century, microscopic tumor cells have been described in the blood of cancer patients [10]. Depending on the testing platform employed, detection rates in early breast cancer patients are reported ranging from 10-90% [11,12,13]. CTC detection generally involves cell enrichment with techniques based on either size or biological characteristics, followed by detection using IHC or molecular techniques for specific biomarkers.

**Figure 1:**
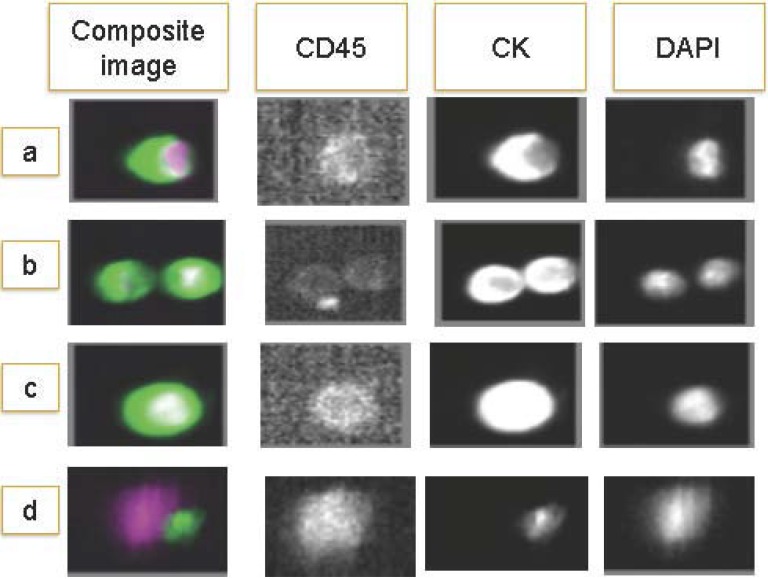
CTC as detected by CellSearch™: differential staining following EpCAM ferrofluid cell capture allows identification of CTC. Staining panel: CD45 leukocyte specific cell surface marker; CK (cytokeratin 8,18,19) epithelial marker; DAPI: (4',6-diamidino-2-phenylindole)detecting nucleic acids. (a–c) CTC: CD45-, CK+, DAPI+; (d) non-CTC, potential leukocyte: CD45+, CK-, DAPI+. (Image provided by the Translational Research Unit, Hospital of Prato).

Many techniques have emerged for the detection of CTC, with diverse methodology, strengths, and weaknesses. See Table 3. The FDA approved semiautomated CellSearch™ system takes advantage of the epithelial cell expression of the epithelial cell adhesion molecule (EpCAM) to enrich the sample [11]. EpCAM antibodies are attached to microscopic iron particles (ferrofluid). CTCs bind EpCAM with subsequent magnetic separation from the blood. For identification as CTCs, these cells must be positive for CK and nucleic acid (DAPI) and negative for CD45, a leukocyte cell surface marker. Histologically, they must be large nucleated cells with visible nucleoli. See Figure 1.

New tools, such as a recently described anti-EpCAM, and flow based microchip technology, ‘CTC-chip’, offer greater sensitivity in cell capture [14]. With this methodology, cell enrichment is achieved by pumping blood continuously through an array of thousands of EpCAM antibody coated microscopic columns. These columns sit on a silicon chip which is the size of a microscopic slide. Captured EpCAM+ cells then undergo staining for expression of DAPI, CK, and CD45, allowing distinction between tumor cells and leukocytes. Advantageously, these intact captured cells are amenable to molecular studies.

Another technique, EPISPOT (EPIthelial ImmunoSPOT), detects only viable cells [15]. Following CD45+ cell depletion, cells are cultured and cellular protein secretion is assessed (e.g., of CK19 and MUC1). Only viable cells will produce and secrete protein at a detectable level. The assay detects protein secretion at a single cell level. This assay allowed detection of viable CTC in 90% and 54% of patients with and without overt breast cancer metastases, respectively. Interestingly, early patients had less CTC, but a higher proportion of the CK19+, MUC1- phenotype. This phenotype has been associated with stem cell-like properties, which may be critical in understanding disease progression [16]. 

**Table 3:** CTC detection: A variety of tools exist for identification of CTC, with variation in enrichment and detection approaches.

Assay System	Enrichment	Detection	Comments
CellSearch™	EpCAM antibody coupled ferrofluid	+ marker: CK, DAPI	Semiautomated system with FDA approval
		− marker: CD45	
EPISPOT	Depletion of CD45+ cells	Secretion of proteins	Detection of viable epithelial secreting cells
		e.g., CK19; MUC1	
CTC-chip	EpCAM-antibody coated microposts	+ marker: CK, DAPI	Captured cells suitable for molecular analysis
		− marker: CD45	
MAINTRAC™	Red blood cells lysis	+ marker: EpCAM	High incidence of positive events.
		− marker: CD45	
Ikoniscope™	Ficoll or filtration	+ marker: EpCAM, CK7/8	Two epithelial specific Ab and FISH
		FISH: cr 7/8	
Ariol™	Red blood cells lysis; CK- and EpCAM-antibody coupled microbeads	+ marker: CK8/18/19	Detection of EpCAM+ EpCAM-CTCs
		− marker: CD45	
RT-PCR methods	Immunomagnetic/gradient	Assay specific mRNA	CTC cannot be morphologically identified
		e.g., CK19, HER2, EpCAM	

## 4. Prognosis

### 4.1. Prognosis and DTC

Detection of DTC at the time of surgery for the primary tumor in stage I-III breast cancer is an independent prognostic factor for poor outcome [7,9,17,18,19,20]. Many studies show correlations between DTC and tumor size, tumor grade, and lymph node status.

Several small studies have shown the poor prognostic significance of DTC in univariate analyses [9,17,18,19]. The largest analysis of DTC in the bone marrow came from a pooled analysis of 4703 patients from 9 prospective studies with 10-year follow-up [7]. The pooled analysis was strengthened by the use of individual patient data. The presence of micrometastases was a significant and independent prognostic factor for poor overall survival, breast cancer specific survival, disease-free survival, and distant disease-free survival. In multivariable analysis for death from breast cancer, DTC were an independent predictor of poor outcome. The median follow-up among survivors was 62 months. For overall survival and breast cancer specific survival, the mortality ratios for micrometastases *vs.* no micrometastases were 2.44 (95% CI 2.08–2.86; p < 0.001) and 2.15 (95% CI 1.87–2.47, p < 0.001).

Despite their clinical validity, there are concerns regarding analytical validity. Assays are heterogeneous and are not clearly standardized or reproducible. The clinical utility of DTC is not clear. Due to the high correlation between DTC and high grade, large tumor size, and nodal involvement, many patients would already be considered candidates for systemic therapy without considering bone marrow status. Conversely, for a patient with an apparently good prognosis with a small, node-negative, low grade tumor, it is unclear that a positive bone marrow result is sufficient to warrant differential recommendations for adjuvant therapy.

### 4.2. CTC Prognosis

The most robust evidence of CTC in breast cancer has emerged from the metastatic setting [21,22,23]. It has been known, for some time, that the presence of circulating tumor cells in advanced breast cancer correlates with a poor outcome. In an early study, 177 women with metastatic disease had CTC measurement prior to commencing systemic therapy. Women with <5 CTC/7.5 mL of blood before therapy had a statistically significantly longer overall survival (OS) and progression free survival (PFS) compared to women with >5 CTC/7.5 mL of blood (OS 18 *vs*. 10 months; PFS 7.0 *vs.* 2.7 months) [21]. Subsequent reports in the metastatic setting confirm CTC levels as a good predictor of overall survival and reveal that persistently high levels of post-treatment CTC correlate with a particularly poor prognosis. 

Whilst the prognostic value of CTC in the metastatic setting has been substantiated, their biological role and clinical significance in early disease remains unclear. In early disease, CTC are found in fewer patients and are lower in number. It is hypothesized that CTC play a role in the metastatic process by breaking away from the primary tumor, circulating in the bloodstream, and establishing secondary tumor deposits in distant sites. 

Data in support of CTC as having prognostic potential is emerging. Stathopoulou *et al.* [24] used RT-PCR to detect CK19 mRNA positive cells. In 148 patients with Stage I and II breast cancer post-surgery and pre-systemic therapy, 30% of patients had a positive result. During the short median follow-up of 28 months, 13% of patents developed metastases and 5% died from breast cancer. Positivity for CK19 mRNA was reported to have a significant independent influence on DFS and OS. Using the same approach, Xenedis *et al.* analyzed 167 patients with node-negative disease pre-initiation of systemic adjuvant therapy [25]. CTC detection was an independent prognostic factor for reduced DFS and OS. The same group assessed the prognostic potential of a panel of three biomarkers by RT-PCR: CK19, mammaglobin (MGB1), and HER2 [26]. From a single institutional tumor biobank, 175 patients who had received adjuvant chemotherapy for Stage I–III breast cancer were randomly chosen. Cells were detected using real time (CK19) and nested (MGB1, HER2) PCR. Seventy-seven patients had at least one marker detected. In univariate analysis, detection of all three markers was associated with shorter DFS. In multivariate analysis, CK19 and MGB1 were associated with shorter DFS.

CellSearch™ was employed by Pierga *et al*. in the neoadjuvant setting to detect CTC pre- and post- neoadjuvant chemotherapy in 97 patients [27]. CTC were detected in about one quarter of patients. This is the first study to show that CTC detection by CellSearch™ has independent prognostic value for early relapse. Using a cut-off of 1 CTC/7.5 mL of blood sample, the presence of CTC pre- or post- chemotherapy, or both, correlated with poor outcome. Of note, median follow-up was only 18 months. Interestingly, the pattern of change of CTC from between the pre- to the post treatment sample did not correlate with the response of the primary tumor. This lack of correlation between response of the primary tumor and CTC to neoadjuvant chemotherapy was also observed by Riethdorf *et al*. in the GEPARQUATTRO study [28], but contrasts with other data from the neoadjuvant setting, in which CTC response mirrored that of the primary tumor [13].

CellSearch™ was also used by Rack *et al.* in a sub-study of the SUCCESS trial [29]. Of 1500 patients with node positive or high risk node negative disease, more than 1 CTC was detected in 146 patients (10%) prior to taxane-based adjuvant therapy and in 130 (9%) patients following adjuvant therapy, with only 15 patients (10%) overlap between the groups. Preliminary results reveal that detection of CTC following chemotherapy was a significant predictor for reduced disease-free and overall survival. Notably, detection of CTC prior to therapy did not correlate with prognosis. Median follow-up at the time of reporting was only 12 months. Longer term follow-up is required to confirm these early findings. 

These results for CTC are encouraging, but they require validation in large multicenter trials with standardized methodology. For CTC, many detection tools exist. Ongoing challenges in the use of CTC include improvement in sensitivity and specificity, standardization of techniques, and evidence of reproducibility.

## 5. Serial Assessment to Monitor Efficacy of Adjuvant Therapy

Detection of micrometastases may play a role in monitoring the efficacy of adjuvant treatment. It would be ideal before commencing systemic therapy to have some degree of certainty of its efficacy. Currently, beyond the HER2, for antiHER2 therapy, and the estrogen receptor, for endocrine therapy, we lack predictive tools for specific therapies. Certainly for chemotherapy, we have no robust biomarkers to guide the use of specific cytotoxic agents. In their absence, early detection of response would be helpful. As compared to the neoadjuvant or metastatic settings, the adjuvant setting does not allow determination of benefit based on tumor measurement, tumor markers, or symptoms. 

Pachmann *et al*. assessed 91 patients in the adjuvant setting. CTC were assessed pretreatment, prior to each chemotherapy cycle, and at the completion of treatment [13]. CTC were detected using the MAINTRAC method, using laser scanning cytometry of anti-EpCAM stained epithelial cells separated from whole blood (without magnetic enrichment or enrichment procedures). CTC were detected in 90% of patients. Of note, the cohort assessed had been selected based on traditional risk factors as candidates for chemotherapy. Three trends in CTC were seen during therapy: a 10-fold or more decrease, marginal change, or a 10-fold or more increase. These patterns of change in CTC were highly predictive of outcomes. Twenty-two percent of patients relapsed within 40 months. Relapses were seen in one of 28 patients with a CTC decrease, five of 30 patients with minimal change, and 14 of 33 with a CTC increase.

This is promising data. CTC’s ease of sampling lends itself to serial testing during adjuvant therapy. Issues are raised, such as the timing and frequency of CTC assessment during therapy. If these results are validated, a challenging issue is what to do in response to a rise in CTC during therapy. Is a rise in CTC reason enough to stop therapy? Options are to continue, change, or stop treatment. Changing treatment would seem to be the logical option. However, results from the neoadjuvant setting, in which chemotherapy was switched due to a non-responding primary tumor, did not prove such a switch to be useful in improving outcomes [30]. This situation is further complicated by neoadjuvant results of a paradoxical rise in CTC during taxane therapy, even when the primary tumor was responding [31]. A trial in which treatment decisions were made based on CTC response would be required to determine their usefulness in guiding therapy. Such a trial is underway in the U.S.A. in the metastatic setting (SWOG S0500).

Detection of micrometastases following adjuvant therapy has also been reported to correlate with a poor prognosis [32]. In a study exploring DTC, 723 patients underwent a bone marrow aspirate at a median of 32 months following primary diagnosis. Of these patients, 15% had detectable DTC. Further follow-up of these patients revealed that, at a median follow-up of 55 months post primary diagnosis, the presence of DTC was associated with worse breast cancer specific survival (RR 3.42 95% CI 1.64–7.13, p = 0.001). Similarly, as already described, the presence of CTC following neoadjuvant and adjuvant chemotherapy has been shown to correlate with poor outcome [27,29]. 

## 6. Biocharacterization of Micrometastases

Over the last decade, with the aid of molecular profiling and multigene tools, we have a much greater understanding of the biological heterogeneity within breast cancer [33]. Between individuals, breast cancer can have marked diversity in behavior, response to treatment, and outcomes. For an individual, intratumoral heterogeneity further complicates the picture. Lack of homogeneity is evident, for example, in patients with focal clones of HER2 positivity within a predominantly HER2- tumor, or within patients with endocrine sensitive primary disease, who develop triple negative metastases. Micrometastases are no exception, with discrepant biological features reported between primary tumor and DTC/CTC. Comparative genomic hybridization showed that DTC in the BM lack genomic aberrations observed in arbitrarily selected areas of the primary tumors [34]. This heterogeneity may explain neoadjuvant results of persistent micrometastases, despite complete pathological response in the primary tumor [35,36]. Biocharacterization of DTC and CTC may define therapy targetable subpopulations.

For example, for a patient with a HER2- primary tumor, detection of HER2+ CTC may worsen the prognosis, but open the therapeutic window for HER2 directed therapy. A study by Ignatiadis *et al*. assessed the HER2 status of the primary tumor and contemporary CTC [26]. Of 48 patients with HER2+ primary disease, 11 had HER2+ CTCs. But of interest, 38 of 124 patients with HER2- primary disease had HER2+ CTC. Such discordance is also reported using the AdnaTest to detect CTC, with greater HER2 positivity and less HR positivity in CTC found, when compared with the primary tumour [37]. Such intratumoral heterogeneity may partially explain reports of trastuzumab benefit beyond the HER2+ population. A subset analysis of the NSABP-B31 trial, one of the key adjuvant trials to show trastuzumab benefit in HER2+ early stage breast cancer, reports benefit of trastuzumab in HER2- patients [38]. Demonstrable intratumoral heterogeneity in early disease may also partially explain findings in the advanced setting, in which HER2 status of CTC may show discordance with that of the original primary tumor [39].

Beyond HER2, biocharacterization of CTC may also influence decisions about adjuvant endocrine therapy and potentially novel targeted agents, such as anti-EGFR agents in TNBC. Such an approach has been shown in lung cancer, in which CTC detected by the CTC chip allowed DNA analysis for specific mutations of the EGFR gene [14]. 

## 7. Challenges with Micrometastases

Many issues regarding micrometastases remain open for debate.

### 7.1. Are These Definitely Cancer Cells?

There are no criteria to unequivocally define cells as malignant, however the evidence strongly supports them as being malignant. Many detection methods depend on identification of epithelial specific markers. However, not all cells that stain positive with epithelial anti-cytokeratins can be unequivocally defined as malignant. Support for these cells as tumor cells is provided by molecular analyses and clinical studies showing their prognostic significance [7,24,25,26].

### 7.2. Are All Breast Cancer Cells Detected?

To be clinically useful, DTC and CTC detection must identify all breast cancer cell types. This is difficult, as there is great diversity in breast cancer biology, coupled with great diversity in detection platforms. Variation in detection rates between techniques raises questions about whether different platforms are detecting different cells. Cytokeratins have become a widely used protein marker for the detection of epithelial tumor cells. With IHC, results depend on both the pattern of CK expression and the CK panel employed by the particular assay. Different antibodies may give different results for the same patient [40]. A false negative result may result from loss of cytokeratin expression (e.g., loss of CK19 expression by cells that have undergone epithelial mesenchymal transition or treatment induced changes in CK expression, in which lack of expression may be incorrectly interpreted as the elimination of disease). 

A recent pre-clinical study specifically assessed the ability of CellSearch™ to detect recognized breast cancer subtypes [41]. *In vitro* genomic profiling confirmed the molecular subtype of 10 breast cancer cell lines. Two cell lines were selected from each of five following subtypes: luminal A, luminal B, normal-like, basal, and HER2. From each cell line, 50–150 cells were injected into normal blood. CellSearch™ did not detect the normal-like subtype. These findings were not confirmed in a subsequent exploratory clinical study involving a limited number of patients (n = 58), in which CTC detection was reported in all subtypes, including normal-like (CTC in 2 of 7 patients) [42]. Normal-like disease may be particularly aggressive with expression of cell markers, particularly with high CD44 and high TWIST.1 expression and low CD24 expression. This is suggestive of their breast cancer stem cell-like properties. Tests that recognize all breast cancer subtypes are required and further studies will address CTC detection across defined subtypes.

### 7.3. Are DTC and CTC the Same Cells?

It is unclear if DTC and CTC are the same cells. If CTC measurements could replace DTC, this would be ideal, due to ease of collection of peripheral blood compared with invasive bone marrow sampling. Two studies, in which patients had both DTC and CTC assessment by IHC, showed a correlation between the two, but a higher rate of detection of DTC [43,44]. This may be explained by the more constant presence of DTC in the bone marrow with sporadic shedding of CTC. However, a more recent study using the AdnaTest for CTC detection reported DTC in 23% of patients and CTC in 16% of patients, but no correlation between the two [36]. Thus with current evidence, CTC cannot be considered a surrogate for DTC. 

### 7.4. Are These Cells Viable?

A critical question is whether these cells are apoptotic cells or whether they are viable cells capable of self-renewal and systemic metastases. If they are viable, with self-renewal properties and the capacity to deposit systemically and progress, they may provide important clinical information in the adjuvant setting. If they are non-viable, their detection may have limited clinical meaning in early breast cancer. In contrast, in metastatic disease, the presence of CTC, viable or not, is shown to predict metastatic disease prognosis [21,22].

## 8. Long-term

**Figure 2:**
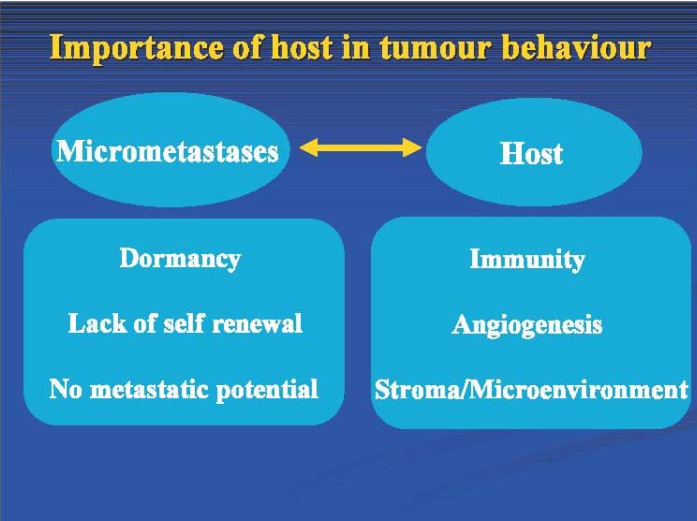
Features important for the consideration of the long-term outcome of patients with detectable micrometastases; note that a substantial number of patients with detectable microscopic disease remain disease-free in the long-term. Features of the tumor, the host, and the interaction between them may be important. Future prognostic tools may incorporate features of both.

The fact that not all patients with DTC or CTC develop clinically detectable metastatic disease [7,45] is of particular interest. About 50% of patients with early breast cancer and detectable bone marrow DTC do not relapse, even after 10 years, whereas relapse occurs in patients without detectable DTC [7]. There is obviously more at play than the presence of detectable cells, as 50% of women with DTC will not develop clinically evident relapse. Detection of micrometastatic disease alone is not enough. This invites exploration of the characteristics of the micrometastatic disease, the individual, and the interaction between the two (see Figure 2). Tumor cells may be dormant, lack autonomous proliferation, and lack metastatic potential. The cells may be rendered dormant by the host stroma. For survival and progression, tumor cells must evade host immunity and be supported by a conducive host environment with high angiogenic potential and a favorable microenvironment. New tools are required to assess this dynamic multifactorial interaction between tumor and host, with identification of biomarkers that predict progression and clinically evident relapses.

## 9. Conclusions

Our current approach for risk assessment and treatment decisions in the adjuvant setting is based on presumption of residual disease. We now have tools to detect DTC and CTC. Despite their prognostic potential, consideration of micrometastatic assessment in routine clinical practice is limited particularly by the lack of standardized reproducible methodologies and the lack of multicenter validation studies. The response of DTC/CTC during adjuvant therapy and the appropriate reactions to these responses require further exploration. Biocharacterization of micrometastases may have important prognostic and therapeutic implications. Incorporation of host features into clinical tools may improve risk assessment for individuals with early breast cancer.
